# Supplementary material for: Are carbon emissions trading and green financial instruments synergistic? -Comprehensive quantitative research based on content analysis
Source: PLoS One. 2024 Mar 7;19(3):e0298601. doi: 10.1371/journal.pone.0298601 (PMC10919601; doi:10.1371/journal.pone.0298601)
Supplement: S1 Data — (ZIP) [file pone.0298601.s001.zip › Data/地级市Z_F工作报告.zip!/╔╜╢1⁄2/╢1⁄2╙¬╩╨/└┤╫╘╣1⁄2╓┌║┼í╛┬φ┐╦╩2╛▌═°í┐_2009.docx]

各位代表：

现在，我代表市人民政府向大会作政府工作报告，请予审议，并请市政协委员和其他列席会议的同志提出意见。

一、2008年政府工作回顾

过去的一年是极不寻常、极不平凡的一年。一年来，在党中央、国务院、省委、省政府和市委的正确领导下，全市上下全面贯彻党的十七大和十七届三中全会精神，深入贯彻落实科学发展观，积极应对国内外复杂多变的经济形势，齐心协力克服困难，奋力开拓扎实工作，黄河三角洲开发建设取得重要进展，经济社会实现了又好又快发展。初步统计，2008年全市实现生产总值2065亿元，比上年增长13.7%；规模以上工业增加值1680亿元，增长14.8%；全社会固定资产投资868亿元，增长24.2%；社会消费品零售总额254亿元，增长23.1%；地方财政收入70.95亿元，增长18%；金融机构各项存款余额1032.8亿元，比年初增加188.3亿元，贷款余额695.9亿元，比年初增加100.6亿元。万元生产总值能耗、二氧化硫和化学需氧量排放继续下降。全面完成了年初确定的各项任务指标。

一年来，我们深入实施黄河三角洲开发建设总战略，着力发展经济，深化改革开放，保障改善民生，促进社会和谐，做了大量富有成效的工作。

(一)积极应对挑战，经济保持平稳较快发展。去年是经济形势复杂多变的一年。年初，国家实行“双防”调控政策，严把土地、信贷闸门和市场准入门槛。进入二季度，又面临煤电油运紧张、流动资金短缺、市场物价波动“三大挑战”。进入十月份以来，国际金融危机对我市经济造成了严重冲击，经济运行面临严重困难和巨大考验。面对煤电油运紧张，油地联手争取增加了15%的用电负荷，积极调运电煤，支持发电企业生产，保障了电煤供应，地方工业用电量增长了13.4%。面对流动资金短缺，召开了两次政银企对接洽谈会，签约贷款金额372亿元，有效缓解了企业资金困难问题。面对市场物价波动，加强物价监管，实行价格临时干预措施，拿出专项资金，搞好群众生活必需品储备，有效地保证了市场供给、价格平稳。面对投资下滑趋势，在全市开展了三次项目集中开工月活动，共开工投资5000万元以上项目119个，总投资529亿元，有效地拉动了经济增长。面对国际金融危机的严重冲击，我们积极应对，快速出手，实行“三个确保、三个加快”，出台了14条政策措施。成立了扩大内需和项目投资工作综合协调领导小组、工业经济运行指挥部，集中帮助企业解决资金、市场等问题，筛选上报了一批重点项目，已经争取到国家和省无偿投资9130万元。由于应对及时，措施超前，使全市经济在困难局面下保持了平稳较快发展。

(二)全力实施突破，迅速拉开黄河三角洲开发建设的框架。去年，在宏观环境趋紧、制约因素增多、困难压力极大的情况下，全市上下凝心聚力，坚定不移地抓推进、求突破，黄河三角洲开发建设取得了良好开局。一是，“四区一城三网”建设取得重大进展。临港产业区，与中海油签订了全面合作协议；港口建设一期工程完工，二期工程前期工作顺利推进，港区配套工程加快实施，一批大项目先后落地。生态旅游区，被评为国家4A级景区，游客中心、游船码头、汽车营地等项目已经开工。现代渔业示范区，4.1万亩起步区基础工程全面展开。高端产业区，生态工业园、软件园和“生态谷”建设快速推进，一批科技含量高、带动力强的大项目先后开工。黄河水城，重点实施了“三园一区一岸线”工程，济南路桥基本建成，会展中心主体工程完工，文化公园广场基础工程基本完成，清风湖景区河湖贯通、广利河岸线改造等工程完成年度任务。“三网”绿化，去年完成投资7.6亿元，绿化面积28万亩，取得明显成效。二是，“三路两港一场”建设加快推进。威乌高速公路建成通车，黄大铁路已开工建设，德龙烟铁路前期工作进展顺利，今年开工。东营港引桥和2个3万吨散杂货泊位建成，2个5万吨液体化工泊位完成立项，今年上半年开工。广利港建设前期准备工作加快推进。东营机场跑道改扩建完工，新的航站楼即将开工建设。三是，大政策争取工作取得突破性进展。黄河三角洲高效生态经济区建设已经上升为国家战略，具体实施规划国家发改委正在论证审批。土地管理改革试点城市、资源型城市可持续发展试点示范城市、生态用水指标、黄河标准化堤防建设等争取工作取得重大进展，正待国家批复。省委、省政府出台了黄河三角洲高效生态经济区发展规划和34条政策措施，并批准东营为发展高效生态经济改革试点市。

(三)大力调整经济结构，产业层次明显提升。加快推进工业结构调整，重点抓了“一突破两优化”。“一突破”，就是突破高新技术产业。发挥东营经济开发区的龙头作用，新上了大规模集成电路、LED、汽车制造等一批项目，现有企业技术改造也取得重要进展，全市高新技术产业产值达到850亿元，增长40%。“两优化”，就是优化提升石化产业，引进中海油整合石化企业，不仅保证了油源，而且为企业技术提升、规模扩大、产业链条拉长创造了条件；优化提升石油装备制造业，举办了中国(东营)国际石油装备与技术展览会，与美国休斯敦市签订了合作协议，搭建起了合作发展平台。大力发展服务业，重点突破文化旅游业，积极争创中国优秀旅游城市并顺利通过验收。现代物流、科技信息等生产性服务业加快起步，商贸餐饮等生活性服务业进一步提升，服务业增加值增长15%。努力改善区域经济结构，推动市属经济与县域经济协调发展，地方经济实力进一步壮大。

(四)着力加强“三农”工作，新农村建设扎实推进。围绕农民增收，大力推进农业产业化，加快构建农业“三次产业”联动发展的格局。粮棉获得丰收，渔业、畜牧业等优势产业加快发展，水产品和肉蛋奶产量分别增长6%和12%。积极发展新兴农业，休闲观光农业园(点)发展到40个。扶持农业龙头企业发展，新增省级以上龙头企业11家。加大农产品品牌培育和推介促销力度，黄河口大闸蟹跻身山东省十大渔业品牌。着力提高农民科技素质，免费培训农民11.3万人。加强农业和农村基础设施建设，实施了一批节水改造和河道治理工程，改造中低产田11.7万亩，中心渔港一期工程完工；新建农村水质检测中心6处，修复改造农村危桥40座，新建农村沼气池7000个，农村生产生活条件进一步改善。

(五)深化改革开放，体制机制创新迈出新步伐。改革投融资体制，“四区一城”全部搭建起了融资平台，市场化运作水平进一步提高；2家企业在海外成功上市。扎实推进农村综合改革，林权制度改革试点进展顺利，强农惠农政策全面落实。深化企业改革，支持油田存续企业改制。金融、价格及社会领域的改革全面推进。不断拓宽对外开放的广度和深度，外经外贸平稳发展，实现进出口总值41.2亿美元，增长55.3%，其中出口19.7亿美元，增长40.7%；吸收外商直接投资1.8亿美元，增长15%。

(六)加大解决民生问题的力度，群众生活进一步改善。千方百计增加城乡居民收入，城镇居民人均可支配收入达到19300元，增长14.6%；农民人均纯收入达到6600元，增长12.5%。加强就业再就业工作，新增城镇就业4.1万人，城镇登记失业率为1.88%。开展城镇居民基本医疗保险试点，参保率达到90%，居全省第一位。建立了物价上涨与提高保障标准联动机制，实行城乡低保人员生活补贴，增加了困难群体冬季供暖补贴；提高了城乡低保、农村五保集中供养、残疾人救助等补助标准。扎实解决困难群众住房问题，新建农村安居房685户，落实廉租房200套，廉租住房货币化补贴410户，建成经济适用房2436套，油田职工住房建设加快推进。支持利津县加快发展，扶持黄河南展区房台建设。加大环保工作力度，推进城乡环境综合整治，人居环境进一步改善。加强食品药品监管，保障了群众饮食用药安全。高度重视手足口病和问题奶粉事件，快速反应，科学防控，对患病婴幼儿采取免费筛查治疗等有力措施，使这两起突发事件在我市得到有效处置。农产品和食品药品安全系统建设、居民小区改造、西城交通环境整治等十件便民实事全部完成。去年，市财政用于民生支出3.3亿元，其中近1亿元用于新增加的民生项目。

(七)切实加强社会建设，保持了社会和谐稳定。加快发展教育事业，完善义务教育经费保障机制，首期农村中小学低标准校舍改造全部完成。实施素质教育，促进基础教育、职业教育协调发展，教育质量进一步提高。加强医疗卫生服务体系建设，建设完善了一批城乡卫生服务设施，方便了群众就医。积极发展文化体育事业，加强城乡文化设施建设，推出了《黄河入海流》等一批文化精品，群众性文化活动蓬勃开展，成功举办了首届黄河口马拉松全国邀请赛和市第八届运动会。毫不放松地抓好人口和计划生育工作，全市人口自然增长率为4.2‰。国防教育、人民防空、民兵预备役和双拥工作进一步加强，外事侨务、民族宗教、对台、档案、统计、史志、无线电管理等工作取得新成绩，妇女儿童、老年人、残疾人权益得到保障，新闻出版、广播电视、红十字会、气象、地震、防灾减灾、社科研究等各项社会事业取得新进步。切实加强精神文明和民主法制建设，思想道德教育、创建文明城市、普法和依法治理等工作深入开展。全面清理行政审批事项，依法行政水平不断提高。扎实开展了维护稳定“百日行动”，圆满完成了北京奥运会和残奥会安保任务。大力推进平安东营建设，全面深化社会治安综合治理，深入开展“打黑除恶”专项斗争，集中开展重点领域专项整治和社会治安综合整治冬季行动，加强出租房屋和流动人口管理，强化基层单位内部安保工作，维护了社会稳定。集中开展了道路交通秩序综合整治，取得了明显成效。强化安全生产监管，开展安全生产大检查，落实整改措施，保持了安全生产形势持续稳定。完善应急管理体系，突发事件应急处置能力进一步提高。

(八)全力支援抗震救灾，取得了重要阶段性成果。四川特大地震发生后，全市上下坚决贯彻党中央、国务院的决策部署和省委、省政府的工作要求，高度重视，迅速行动，全力以赴支援四川抗震救灾。累计捐款捐物1.27亿元，向灾区派出救援队伍635人次，建设过渡性板房1702套，抗震救灾及对口援建支出6967万元。扎实做好对口援建坝底乡工作，编制完成了坝底乡灾后重建规划，确定了援建项目和实施方案，首批项目正在组织实施。

(九)加强油地军校团结合作，形成了团结发展的良好局面。油地军校精诚团结、真诚支持、坦诚相待，一个目标、一条心、一股劲，齐心协力共建美好家园。胜利油田、石油大学、济军生产基地积极参与全市经济社会发展和黄河三角洲开发建设，作出了重要贡献。油地军校深入融合、团结发展，凝聚起无坚不摧的强大合力。

回顾2008年，我们充满自豪。今天的东营，已经成为黄河三角洲开发建设的主战场，正在向着现代化的黄河三角洲中心城市大步迈进。既然，我们能够在风雨中展开腾飞的翅膀，那么，任何困难也阻挡不住我们继续前进的脚步。只要全市上下、油地军校艰苦奋斗，团结包容，百折不挠，拼搏进取，我们的目标就一定能够实现，明天的东营，必将成为黄河三角洲上一颗璀灿的明珠！

各位代表、同志们！过去一年的成绩，是在克服重重困难，经受严峻考验的情况下取得的，来之不易。这是省委、省政府和市委正确领导的结果，是市人大及其常委会监督支持、人民政协和各民主党派参政议政的结果，是全市上下、油地军校、社会各界共同努力的结果。在此，我代表市人民政府向全市人民和社会各界，向所有为东营的建设和发展作出贡献的同志们，向海内外所有关心、支持黄河三角洲开发建设的朋友们，表示衷心的感谢并致以崇高的敬意！

在充分肯定成绩的同时，也要清醒地看到，发展中还存在不少矛盾和问题。主要是，产业结构不够合理，调整优化的任务仍然很重；节能减排形势严峻，环境保护的压力仍然很大；改革创新力度不够，经济运行和社会管理中体制性机制性矛盾非常突出；一些民生问题还没有得到很好解决，部分群众生活仍然比较困难；影响社会和谐的因素较多，维护稳定的任务十分艰巨；有些政府部门效率低下、服务不到位，政府自身建设仍需加强。在今后的工作中，我们一定要高度重视这些问题，加大力度，认真解决。

二、面临的形势和2009年政府工作的任务

2009年，形势严峻，压力巨大，对此我们必须有充分的估计。目前，这场上世纪大萧条以来最严重的国际金融危机还在扩散和蔓延，对全球实体经济的影响仍在进一步加深，其严重后果还会进一步显现。受其影响，我市经济运行中的压力明显加大。企业运行遇到空前困难，市场萎缩，出口受阻，效益下降，有些企业出现了停产半停产；财政减收增支因素大大增加，收支矛盾异常突出；企业减员待工人员不断增加，城镇新增劳动力规模扩大，就业形势十分严峻；农产品价格全面回落，农民工回流，对农民增收造成很大影响。

我们既要充分看到困难，也要坚定信心，充分看到发展面临的机遇和有利条件。一是，国家实施积极的财政政策和适度宽松的货币政策，扩大内需，拉动经济增长，而我们实施的黄河三角洲开发建设的重大工程，完全符合国家的投资方向，并且项目已经开工，恰逢其时，这为我们争取国家财政支持提供了机遇。二是，我市投资环境有了很大改善，特别是立体大交通框架的初步构建，为大投入、大开发、大开放创造了有利条件。三是，经过25年的发展，我市的综合实力明显增强，企业的装备水平不断提升，产业形成了一定规模，保持经济平稳较快增长的基础比较牢固。因此，我们务必坚定必胜信心，扎实做好工作，迎接各种挑战，化危为机，迎难而上，在困难中开拓新的发展空间。

今年是推进“十一五”规划顺利实施的关键一年，也是加快黄河三角洲开发建设的重要一年。做好今年的工作，对于有效应对国际金融危机冲击，维护改革发展稳定大局，顺利实现黄河三角洲开发建设第一步战略目标至关重要。我们要全面贯彻党的十七大和十七届三中全会精神，深入贯彻落实科学发展观，立足扩大投资保持经济平稳较快增长，全力推进黄河三角洲开发建设，加快经济结构调整，转变发展方式，深化改革开放，着力改善民生，促进社会和谐稳定，推动经济社会又好又快发展。

今年经济社会发展的主要目标是：

——经济保持平稳较快增长。全市生产总值增长11%，地方财政收入增长8%。保运行、保增长的措施有效落实。一批重大项目开工建设，全社会固定资产投资增长20%。

——黄河三角洲开发建设实现新的突破。四大主体产业区基础设施配套加快推进，项目进区实现重大进展；黄河水城主体工程基本完成；“三网”绿化拉开主体框架，形成明显的生态景观；立体交通体系初步形成；大政策争取工作取得更大突破。

——经济结构更加优化。大力引进战略合作伙伴，整合提升传统产业。高新技术产业和服务业比重进一步提高。渔业、畜牧业等优势产业规模进一步扩大，农产品加工增值率明显提高。

——发展的协调性增强。经济与文化融合发展，文化旅游业发展实现新的突破。坚持陆海统筹，海洋经济取得新发展。城乡统筹发展，农村改革发展的力度加大。经济与社会协调发展，社会事业全面加强。人与自然和谐发展，人口自然增长率控制在6.5‰以内，万元生产总值能耗降低5.74%，二氧化硫排放量削减15%，化学需氧量排放量削减5%。扩大内需与对外开放相互促进，社会消费品零售总额增长16%，外贸进出口增长10%，吸收外商直接投资增长8%，对外合作迈出新的步伐。

——民生得到进一步改善。就业形势基本稳定，社会保障体系更加完善。城镇居民人均可支配收入增长7%，农民人均纯收入增长7%，城镇登记失业率控制在2.4%以内，居民消费价格总水平涨幅控制在4%以内。同时，做好对口支援坝底乡灾后重建工作，完成上级安排的援建任务。

面对当前形势，实现今年的发展目标，必须更加深入地贯彻落实科学发展观，认真执行国家宏观调控政策，把保增长、扩内需、调结构、重民生、促和谐更好地结合起来；必须更加准确地把握形势，超前预测，积极应对，不断提高驾驭能力和水平，在克服困难中实现新的发展；必须更加自觉地维护大局，加强团结，充分调动方方面面的积极性，形成攻坚破难的强大力量。只要全市上下积极作为，沉着应对，科学务实，共克时艰，我们就一定能克服一切艰难险阻，夺取全市经济社会发展的新胜利！

三、努力保持经济平稳持续发展

应对今年的经济形势，必须打好扩大投资的突破战、企业运行的保卫战、结构调整的攻坚战“三大战役”。打好这“三大战役”，就能保持全市经济平稳持续发展。

(一)打好扩大投资的突破战，拉动全市经济平稳较快增长。扩大投资是本轮国家宏观调控的核心，是当前拉动经济增长的主要动力，是加快推进黄河三角洲开发建设的迫切需要，必须紧紧抓住这个“牛鼻子”。今年全市固定资产投资确保突破1000亿元。一是突出抓好重大项目建设。加快推进已开工项目建设，尤其对34个中央投资项目，要落实配套资金，倒排工期，加快推进，确保今年3月底前形成实物工作量。继续做好项目争取工作，把筛选出的37个重大项目作为重点，加大争取力度，力争有更多的项目列入国家支持计划。同时，积极做好其他各类项目的争取工作。二是切实把握好投资方向。重点投向重大基础设施、“三农”、高新技术产业、现代服务业、民生工程、节能减排、生态保护等重点领域和薄弱环节。三是广开融资渠道，构建多元化的融资体系。积极做好国债资金争取工作，并努力争取省里的支持。扩大财政投资规模，引导企业和社会加大投资。强化金融保障，鼓励金融机构创新信贷方式，增加信贷规模，扩大信贷投放。四是加强投资管理。规范项目运作，严格控制“两高”和产能过剩行业投资，坚决防止低水平重复建设和盲目投资。严格资金监管，坚决防止浪费建设资金，切实提高投资质量和效益。

(二)打好企业运行的保卫战，支持企业保生存、渡难关、求发展。企业是经济之源、发展之本。必须把保持企业健康运行作为最现实、最重要、最紧迫的任务，千方百计帮助企业解决困难，努力争取资金不断链、生产不停工、市场不丢失、职工不下岗，奋力渡过难关。要加大对企业的扶持力度，重点在企业融资、市场开拓、技术改造、减轻负担方面做好工作、搞好服务。市县财政出资设立中小企业互助资金，完善中小企业信用担保体系，搞好小额贷款公司试点工作，提高中小企业信贷资金比重，解决企业资金困难。安排专项资金支持中小企业开拓市场，提高中小企业产品的政府采购比重。落实企业税收优惠政策，全面清理和规范各种收费，对困难企业最大限度地实行“减、免、缓”，严厉查处乱检查、乱收费、乱罚款、乱摊派等行为，切实减轻企业负担。引导企业树立应对困难和挑战的坚定信心，苦练内功，加快科技创新，优化产品结构，提高产品质量，改革营销方式，降低生产成本，提高市场竞争力和抗风险能力。

(三)打好结构调整的攻坚战，加快构建现代产业体系。结构调整是提高经济竞争力的关键举措，也是应对国内外环境变化、实现可持续发展的根本出路。必须加大力度，实现更大突破。一是以机械电子和新能源产业为突破口，大力发展高新技术产业。加快推进大规模集成电路、汽车制造等项目建设，膨胀机械电子产业规模。加快推进太阳能电池、LED、风电和燃气发电装备制造等项目建设，带动新能源产业实现突破。同时，积极发展新材料、生物医药等产业。重点培育30家骨干高新技术企业。二是以提升技术装备水平为突破口，大力改造传统产业。引导企业用好国家增值税转型等政策，积极引进新技术、新工艺、新设备，加强技术改造，推动转型升级，提高竞争力。设立专项资金，扶持企业技术改造，全市企业技改投入要占到工业投入的50%以上。继续推进石油化工、石油装备、橡胶轮胎、纺织服装等传统产业的整合，重点推动中海油加快石化产业整合步伐，力争在石油装备制造业和橡胶轮胎业整合上取得明显进展。三是以文化旅游业为突破口，大力发展服务业。旅游业以“一区一带五湖十园”为重点，配套完善基础设施，整合现有旅游资源，推出一批精品旅游线路。制定文化产业发展规划，扶持优势项目建设，壮大文化产业规模，加快文化与旅游产业的融合。加快发展现代物流业，加强物流园区建设，引进和培育一批物流企业。积极发展服务外包业，加快软件服务平台建设，引进一批软件企业。促进房地产业健康发展，鼓励居民购房，引导和支持房地产企业积极应对市场变化，保持合理的房地产开发规模。同时，加快商贸餐饮、社区服务等产业发展。把发展服务业与扩大消费结合起来，努力消除制约消费的制度和政策障碍，改善居民消费预期，引导和促进居民扩大消费需求。四是以培育特色产业为突破口，大力发展县域经济。支持县区发挥优势，以经济园区为载体，突出产业特色，着力打造支柱产业。制定完善促进县域经济发展的政策措施，加大“突破利津”力度，壮大县域经济实力。

在当前严峻的经济形势下，节能减排工作不能有丝毫放松。要大力推进节能降耗，严格新上项目节能评估和审查，落实差别电价和超耗能加价政策，淘汰落后产能，加快发展循环经济。集中开展国有土地清理整顿，提高土地集约节约利用水平。加强小清河、广利河等重点流域和化工企业集中区等重点区域污染治理，强化重点企业污染治理，在全市石化、盐化行业全面实施污水深度处理和资源利用工程。加大环境执法监察和监测力度，严肃查处环境违法行为。

四、加快推进黄河三角洲开发建设

黄河三角洲开发建设，由于我们不等不靠，快速启动，以干促批，引起了各方面高度重视和广泛认可，已经上升为国家战略。我们必须坚定不移，乘势而上，加快推进。黄河三角洲开发建设虽然是一项长期的任务，但必须树立强烈的紧迫感和使命感，以时不我待、只争朝夕的精神，快干快突，努力形成快速崛起之势。在新的一年里，我们必须继续牢牢抓住这个总战略、总抓手，努力实现新的更大的突破。

(一)在“四区一城三网”建设上实现新突破。四大主体产业区是构建东营现代产业体系最重要的支撑平台，今年的主要任务是尽快落地建设一批大项目，拉开产业框架，打造产业集群。临港产业区，推动中海油加快建设原油储备基地、成品油仓储区、物流园区和中海油工业园。加大投入，推进基础设施配套建设，尽快提高承载能力。完善政策措施，推动“工业北移”，重点推动化工产业向港区聚集。生态旅游区，年内建成游客中心、游船码头、汽车营地等旅游服务项目，规划建设黄河口湿地公园，适时启动黄河口休闲旅游度假区建设，全力打造山东生态旅游龙头景区。现代渔业示范区，加快配套完善基础设施，完成起步区基础骨干工程，建设研发中心，引进大型加工龙头企业，建设高标准养殖示范基地，尽快形成规模。高端产业区，重点推进生态工业园建设，规划建设东营国际新能源产品及装备制造基地。加大在建项目的推进力度，确保早投产、早见效；加大招商力度，加快引进落地一批大项目、好项目；加大投融资力度，增强自我发展能力；加大自主创新力度，尽快把东营经济开发区打造成黄河三角洲高端产业的聚集区。黄河水城，今年是实施广利河综合治理三年规划的最后一年，要加快进度，基本完成预期目标。建成会展中心并投入使用，加快清风湖改造，完成老广蒲河、五六干合排综合治理。加大控制性详规编制和城市设计力度，实现中心城近期建设用地控规全覆盖。加快完善供水供热供气、污水及垃圾处理等市政公用设施。加快完善城市公交体系，积极开展中心城轻轨交通前期工作。同时，加强县城和小城镇规划建设，深入开展城乡环境综合整治，改善城乡面貌。“三网”绿化，突出抓好威乌高速公路等22条路域绿化，全市主要交通干线基本实现全路域绿化，确保完成绿化面积16万亩。

(二)在“三路两港一场”等重大基础设施建设上实现新突破。加快黄大铁路建设进度，开工建设德龙烟铁路，规划论证建设东营港疏港铁路、东营经济开发区铁路和兴广铁路至黄大铁路连接线。开工建设东营港2个5万吨液体化工泊位，力争2010年底前投入使用；口岸联检机构进驻港区办公，实现开港通航。加快推进广利港前期准备工作，确保防沙堤、航道疏浚、码头及水电路讯等各项工程全面开工。加快飞机场航站楼及配套工程建设，上半年完成飞行区工程。加快公路建设，开工建设飞机场至自然保护区一级公路，积极做好疏港公路、东滨高速公路前期规划论证工作。建成东营区段防潮堤，开工建设东营港经济开发区防潮堤。加强黄河入海流路治理，实施清八治理工程。加强能源建设，重点实施大唐国际火电、东营经济开发区热电、胜利电厂扩建和一批风电项目，完善电网设施。深入推进“数字化东营”建设，提高信息化水平。

(三)在大政策争取上实现新突破。努力做好工作，争取国家尽快把我市列为土地管理改革试点城市和资源型城市可持续发展试点示范城市，争取把东营经济开发区升格为国家级经济技术开发区，争取国家批准设立黄河三角洲开发基金、黄河三角洲发展银行、黄河三角洲开发担保公司。继续做好省委、省政府出台的34项政策措施的争取工作，力争取得最大成效。同时，用足用好各项优惠政策，为黄河三角洲开发建设提供强大的政策支持。

五、加快农村改革发展步伐

农业、农村、农民问题，始终是关系经济社会发展的根本性问题，必须作为全部工作的重中之重，按照党的十七届三中全会部署，坚持工业反哺农业、城市支持农村和多予少取放活方针，加大统筹城乡发展的力度，促进农业增产、农民增收、农村繁荣。

(一)大力发展现代农业。按照高产、优质、高效、生态、安全的要求，在稳定粮食生产的同时，大力培植五大主导产业，集中建设一批优势农产品基地。突出发展渔业和畜牧业。搞好黄河口大闸蟹核心生产基地建设，实施海水养殖池塘开发和“上农下渔”池塘整理项目。规划建设黄河三角洲生态畜牧业基地，完善提升十大畜牧龙头养殖园区，新建提升标准化畜牧养殖小区100个。积极发展休闲观光农业，重点建设15个基地。加快培育农业龙头企业，重点支持成长性好、带动能力强的龙头企业发展，拉长产业链条，促进农产品转化增值。培育壮大农村专业合作经济组织，新建一批农民专业合作社，提升农业产业化水平。加强农产品品牌培育和宣传推介，加快农副产品综合批发市场建设，完善农产品流通体系，构建营销网络，提高竞争力。充分发挥政府在农资价格监管和农产品销售中的调控作用，努力减小市场波动对农民收入的不利影响。同时，大力发展农村二三产业，拓宽农民增收渠道。加大农技推广力度，重点推广10项农业技术，强化农业科技信息服务。加强农民科技培训，提高农民科技素质。继续实施放心农资网络工程。加强农业标准化和农产品质量安全工作，建设一批放心农产品基地，强化农产品质量检验检测。加强动植物疫病防控。

(二)改善农村生产生活条件。推进农田水利基本建设，实施宫家闸等引黄工程建设改造，搞好麻湾总干等3条灌渠节水改造，建设一批农田节水工程，治理神仙沟等6条排河，除险加固水库2座，改造中低产田12万亩。实施利北和淄河中游风沙片综合治理。推进农村电网建设，新建改造农村变电站16座。建设改造农村公路300公里。加强农村清洁能源建设，新增沼气用户8000户。实施农村饮水巩固工程，解决8.4万农村人口饮水安全问题。扩大“万村千乡”市场工程和“两进”工程覆盖面，实施放心粮油食品工程和农户科学储粮工程。加强村庄规划改造，改善农村面貌。

(三)深化农村改革。要赋予农民更加充分而有保障的土地承包经营权，加强土地承包经营权流转管理和服务，允许农民按照依法自愿有偿原则，发展多种形式的土地规模经营。深化农村产权制度改革，积极探索农村集体经济的有效实现形式。全面启动林权制度改革，加快建立现代林业发展机制。推进农村金融体制改革和创新，加快培育农村金融体系，鼓励更多的信贷资金投向农业和农村。加快推进农场改革，增强发展活力。全面落实各项强农惠农政策，减轻农民负担，调动农民的生产积极性。加大对“三农”的财政支持力度，建立财政投入稳定增长机制。

六、大力推进改革开放和科技创新

深化改革、扩大开放、科技创新是保增长促开发的三大动力，必须加大推进力度，着力突破体制性机制性障碍，破解发展难题，增创发展新优势。

加快推进各项改革。一是全面启动发展高效生态经济改革试点工作。选择部分重点区域和县区率先开展试点。二是深化投融资体制改革。完善政府投资体制，加快推行代建制。完善“四区一城”融资平台运营机制，拓宽融资渠道，提高融资能力。推进金融改革，组建农村商业银行，吸引股份制银行到我市设立分支机构，争取设立村镇银行。支持发展保险业务。三是积极推进财税体制改革。加快建设公共财政，努力增收节支，强化税收和非税收入征管，大力压减一般性支出，集中财力办大事。完善部门预算、国库集中收付、政府采购等制度，提高财政精细化管理水平。全面实施增值税转型改革，认真贯彻新修订的增值税以及营业税、消费税暂行条例。四是加快行政管理体制改革。推进政府机构改革，切实转变政府职能。深化行政审批制度改革，扩大县级政府经济社会管理权限。推进事业单位综合配套改革。五是积极推进公用事业改革。加快城市供气、供热、公交、污水和垃圾处理等领域市场化改革的步伐，形成合理的市场价格机制。六是加快推进城市管理体制改革。坚持条块结合，重心下移，统筹协调，合理分工，提高城市管理水平。七是继续推进企业改革。推动优质企业上市。增强民营经济发展活力，促进民营经济发展壮大。支持油田存续企业改制，积极争取解决改制企业的土地产权问题。

进一步扩大对外开放。加大外经外贸外资工作力度，提高开放型经济水平。大力开展产业招商、专业招商，围绕“四区一城”和经济结构调整，引进一批“大、高、外”项目。帮助企业克服国际金融危机对出口的影响，调整出口产品结构，培育出口自主品牌，保持出口稳定增长。实施出口市场多元化战略，千方百计巩固老市场，积极开拓新市场。落实促进外贸发展的政策，加强对橡胶轮胎、石油装备、纺织服装、农产品等重点出口企业的扶持。积极扩大进口，增加急需的先进技术、关键设备、重要能源原材料进口。引导有条件的企业积极寻求海外投资机遇，加快“走出去”步伐。加强区域经济合作，积极参与环渤海经济圈和半岛城市群的合作，加强与周边城市的合作与交流。

提高科技创新能力。加大投入，加快建设高效生态经济技术创新示范区、大学科技园高新技术园、可持续发展研究院，打造区域科技创新龙头。健全以市场为导向、企业为主体、产学研相结合的技术创新体系，重点支持企业研发中心建设，大力开发拥有自主知识产权的产品和技术，加快科技成果的转化应用。年内新建省级以上研发机构10家，转化重大科技成果50项。争创国家知识产权示范城市。深入实施人才强市战略，加大人才引进、培养、使用力度。积极引进一批高层次创新创业人才，规划建设院士工作站，吸引院士来我市研发创业；用好职业技术教育资源，搞好校企合作，加快培养培训一批高技能人才；努力创造人尽其才、才尽其用的良好环境。

七、改善民生促进社会和谐

民生问题事关改革发展稳定全局，越是在经济困难的时候，越要高度关注民生，不断改善民生，促进社会和谐。

(一)着力保障和改善民生。就业是民生之本。面对严峻的就业形势，必须采取积极有效的措施，在保证经济健康运行的基础上，努力实现全市就业形势基本稳定。高度重视企业职工待工、下岗问题，政府要发挥好职能作用，全面落实积极的就业政策，进一步完善再就业帮扶机制和就业服务体系，更好地发挥人力资源市场的作用，扩大就业规模；企业要积极履行社会责任，努力做到限产不裁员；加强对放假待工人员的培训，政府给予补贴。统筹做好各类群体的就业工作，实行政府购买公益性岗位安置就业困难人员。鼓励自谋职业和自主创业。完善社会保障救助体系，做好城乡弱势群体、困难群众基本生活的保障工作。扩大城镇居民医疗保险覆盖面，完善农民医疗保险办法和新型农村养老保险制度，提高企业退休人员基本养老金水平，提高优抚对象等人员抚恤和生活救助标准。积极发展社会福利和慈善事业。扩大保障性安居工程建设规模，年内开工建设廉租住房500套、经济适用住房1700套，发放廉租住房货币化补贴1800户，支持油田职工住房建设。继续实施黄河南展区房台建设，新建农村困难群众安居房1200户。加快城市旧住宅小区和棚户区改造。实施提高农民医疗保险水平、改善城市居民饮用水水质等十件便民实事，确保按期完成。

(二)加快发展教育卫生等社会事业。坚持教育优先发展战略，以义务教育为重点，促进各级各类教育协调发展。加大投入改善办学条件，实施中小学校舍抗震安全改造、教学仪器更新和“两热一暖一改”试点工程，加快城区义务教育阶段学校建设，推进实施市技术学院迁建改造，完成东营职业学院教学楼建设。深化教育改革，推进素质教育。加强教师队伍建设。完善以政府投入为主的义务教育经费保障机制。以人人享有基本医疗卫生服务为目标，加强城乡医疗卫生服务体系建设。建成并启用市人民医院病房楼，推行政府购买城市社区公共卫生服务，实行社区100种常用药品“零差率”销售，建成11处城市社区卫生服务中心、35处城市社区卫生服务站。加强乡村卫生设施和医疗队伍建设，搞好县级医院和11处乡镇卫生院建设改造；从今年起利用3年时间，实施600所村卫生室业务用房整修、基本设备配置和乡村医生培训，全面提升农村卫生机构服务能力。加强医德医风建设，提高医疗服务质量。抓紧抓好人口与计划生育工作，切实稳定低生育水平。加强国防教育，抓好民兵预备役建设，巩固扩大双拥共建成果。大力发展残疾人事业，积极创建全国残疾人工作示范城市。关心支持妇女儿童事业，做好老龄工作。进一步做好外事侨务、民族宗教、人民防空、无线电管理、档案、气象、对台、地名、史志、红十字会、防震和减灾救灾等工作。

(三)加强和谐文化和民主法制建设。文化是城市软实力的集中体现。要以打造黄河口文化品牌为目标，以建设和谐文化为着力点，大力发展文化事业和文化产业，推进文化创新发展。尽快建成一批标志性文化设施，加快建设广电中心大楼。加强社区和乡村群众文化体育场所建设，着力推进广播电视户户通、基层综合文化站、农家书屋建设，继续开展电影下乡工作。加强文艺精品创作。深化文化体制改革，培育文化龙头企业，激发文化发展活力。加强文化市场监管，规范文化市场秩序。举办第二届黄河口旅游文化博览会、马拉松全国邀请赛，承办好全国十一届运动会有关赛事。加强社会主义核心价值体系建设，广泛开展群众性精神文明创建活动，深入推进“文明城市”创建，提高全社会文明程度。大力推进依法治市，深入开展普法教育，提高全民法律素质。各级政府要自觉接受人大及其常委会的监督，认真执行人大及其常委会的决议决定，支持人民政协履行职能，认真办理人大代表建议和政协提案。加强与各民主党派、工商联、人民团体和社会各界的联系。扩大基层民主，保障群众依法行使民主权利。

(四)切实加强社会管理。采取综合措施，标本兼治，建立完善维护和谐稳定的长效机制。加强城乡社区建设，打牢社会管理的基层基础。进一步加强出租房屋和流动人口综合管理。完善社会矛盾纠纷排查调处和防范化解机制，加强和改进信访工作，切实做好重信重访等突出信访问题的化解工作，妥善处理人民内部矛盾。深入开展以“打黑除恶”为龙头的严打整治斗争，加强油区治安综合治理，推进“平安城市”基础项目建设，加快城乡警务室建设，提高基层单位内部安全保卫能力，配套完善居民小区物防、技防设施和保安巡防力量，积极推行农村治安保险，广泛开展平安创建活动，努力维护良好的社会治安秩序，保障人民群众生命财产安全。切实加强安全生产，严格落实安全生产责任制，突出重点领域，加大安全投入，加强安全教育，强化监督检查和隐患排查整改，坚决遏制重特大安全事故的发生。高度重视食品药品安全工作，加强生产、加工、流通等各个环节的安全监管，严厉打击制假售假行为。健全应急管理体制机制，提高应对突发事件和风险的能力，保障公共安全。

八、进一步加强政府自身建设

面对严峻的形势和挑战，必须进一步加强政府自身建设，着力提高政府的创新力、执行力和公信力，为促进经济平稳较快发展、加快推进黄河三角洲开发建设提供坚强保障。

(一)坚持解放思想，开拓创新。解放思想是克难致胜的法宝，越是在困难的时候越要解放思想、开拓创新、勇于进取，闯出一片新天地。要始终保持良好的精神状态，坚定信心，勇于面对困难，勇于面对挑战，敢于迎难而上，拼搏进取，奋发有为。善于洞察和把握国内外发展大势，未雨绸缪，善于危中寻机，抢占先机，赢得主动。必须切实提高驾驭复杂局面的能力、破解难题的能力、科学运作的能力，以创新的精神和改革的办法，奋力突破困境，推动发展。认真开展深入学习实践科学发展观活动，务必在转变思想观念、提高创新能力、解决突出问题、推动科学发展上取得实效。

(二)坚持维护大局，形成合力。严峻的形势和艰巨的任务，不仅是对意志和智慧的考验，而且是对我们整体凝聚力的考验。在事关生存和发展的重要关头，我们必须更加自觉地维护大局，以大局统一思想、指导行动，集中精力谋发展，一心一意干事业。政府各部门要牢固树立全市“一盘棋”的思想，正确处理局部与全局的关系，站在大局思考问题，围绕大局干好工作，加强配合，协调联动，坚决克服各自为政、推诿扯皮等问题。要倍加珍视团结，齐心协力，共渡难关。要严明纪律，切实做到有令必行，有禁必止，确保政令畅通。

(三)坚持求真务实，狠抓落实。克服困难要靠艰苦奋斗，推进黄河三角洲开发建设要靠真抓实干。全市上下务必弘扬求真务实的精神，求科学发展之真，务东营发展之实；求形势变化之真，务克服困难之实；求黄河三角洲开发建设之真，务加快推进之实。进一步改进工作作风，深入实际、深入基层、深入群众，做到问政于民、问需于民，尊重群众，顺应民意。进一步改进会风文风，减少应酬性活动，集中精力抓好工作落实。完善目标责任制，加强督查考核，确保各项工作落到实处。加快建立行政问责制，有责必问，有错必究，努力建设责任政府。

(四)坚持执政为民，廉洁勤政。我们的政府是人民的政府，执政为民是各级政府的崇高使命。全体政府工作人员要时刻把人民群众的冷暖安危放在心上，努力为群众办实事、解难题。大力推进依法行政，实行科学民主决策，深化政务公开，提高行政效率，为人民掌好权、服好务。大力提倡艰苦奋斗，坚决反对奢侈浪费。加大专项治理力度，重点解决环境保护、食品药品安全、安全生产、土地征收征用和房屋拆迁等方面群众反映强烈的问题，坚决纠正损害群众利益的不正之风。把反腐倡廉建设放在更加突出的位置，完善惩治和预防腐败体系，着重加强对建筑工程招投标、土地使用权交易、政府采购、政府投资项目管理等重点领域的监管。严肃查处各类违法违纪案件，依法严惩腐败分子。

各位代表、同志们！油地军校团结结合始终是东营的巨大优势，始终是推进黄河三角洲开发建设的强大力量。合则强大，和则成功。在任何时候、任何情况下，都必须高举油地军校团结发展的旗帜，牢固树立大市民观念，同呼吸共命运、一条心一股劲，并肩携手，和衷共济，推进改革，谋求发展，共同建设我们美好的家园，共同开创黄河三角洲美好的未来！

各位代表、同志们！困难激励斗志，开拓必有收获。让我们更加紧密地团结在以胡锦涛同志为总书记的党中央周围，坚持以邓小平理论和“三个代表”重要思想为指导，深入贯彻落实科学发展观，在省委、省政府和市委的坚强领导下，进一步解放思想，抢抓机遇，克服困难，勇往直前，奋力谱写黄河三角洲开发建设的新篇章，以优异成绩迎接新中国成立60周年！
